# Adverse Events of Prostacyclin Mimetics in Pulmonary Arterial Hypertension: A Systematic Review and Meta-Analysis

**DOI:** 10.3390/jcm8040481

**Published:** 2019-04-09

**Authors:** Christina Picken, Konstantinos C. Fragkos, Mohammad Eddama, Gerry Coghlan, Lucie H. Clapp

**Affiliations:** 1 Institute of Cardiovascular Sciences, University College London, 5 University Street, London WC1E 6JF, UK; christina.picken.14@ucl.ac.uk; 2 University College London Hospitals, 250 Euston Road, London NW1 2PG, UK; constantinos.frangos.09@ucl.ac.uk; 3 Division of Surgery and Interventional Sciences, University College London, 21 University Street, London WC1E 6AU, UK; eddama@doctors.org.uk; 4 Department of Cardiology, Royal Free Hospital, London NW3 2QG, UK; Gerry.coghlan@nhs.net

**Keywords:** pulmonary arterial hypertension, prostacyclin, IP receptor, treprostinil, selexipag, iloprost, beraprost, adverse events, meta-analysis

## Abstract

Prostacyclin mimetics (PMs) are effective for the treatment of pulmonary arterial hypertension (PAH). However, their clinical use may be limited by their adverse events. This study aims to quantify the different PM adverse events (AEs) with regard to their selectivity towards the prostacyclin (IP) receptor and their administrative routes. The study included randomised, placebo-controlled trials comparing iloprost, beraprost, treprostinil, and selexipag to placebo (published 2002–2016). We report the group efficacy differences between treatment and placebo by weighted and standardised mean difference. The probability of adverse events was determined by the odds ratio (OR). Of the 14 randomised clinical trials involving 3518 PAH patients, outcome and adverse event data were meta-analysed by drug type and route of administration. Prostacyclin mimetics comparison demonstrated a more significant discontinuation of the IP-selective agonist, selexipag, due to an adverse event (OR = 2.2; 95% CI: 1.5, 3.3). Compared to placebo, site pain associated with subcutaneously administered treprostinil was the most significant likely adverse event (OR = 17.5; 95% CI: 11.1, 27.1). Parenteral PMs were associated with fewer adverse effects overall. The overall efficacy of PMs to improve 6-minute walk distance by 16.3 meters was significant (95% CI: 13.0, 19.7). Decreases in pulmonary vascular resistance index (SMD = −5.5; 95% CI: −10.1, −0.9; *I*^2^ = 98%) and mean pulmonary arterial pressure (SMD = −1.0; 95% CI: −2.6, −0.7; *I*^2^ = 99%) in treatment groups were found to be significant. Adverse event profiles varied in response to administration route and PM type but were not negated by use of a selective IP agonist. Prostacyclin mimetics exposure to non-target IP receptors may underpin some AEs reported.

## 1. Introduction

Pulmonary arterial hypertension (PAH) is a rare but life-limiting inflammatory remodelling disease of the small (<500 µm) pulmonary arteries [1]. Pulmonary vascular resistance progressively increases, ultimately leading to right ventricular failure. Among other biological targets, the prostacyclin pathway is therapeutically targeted by the administration of synthetic prostacyclin (epoprostenol) and its stable analogues, such as iloprost, treprostinil, and beraprost, which potently bind to prostacyclin’s native IP receptor, with a K_i_ of 2, 4, 32, 39 nM, respectively [2]. These compounds have a range of biological actions, promoting vasodilatory, anti-proliferative and anti-thrombotic effects in the pulmonary circulation [3,4,5,6,7,8]. However, they are also known to activate other prostanoid receptors, some of which could contribute to therapeutic efficacy (DP_1_, EP_2+4_), whilst others may have a negative impact (EP_1+3_, TP) [2,4]. Of note, compared with the prostacyclin (IP) receptor, iloprost is equipotent at the EP_1_ receptor, treprostinil 10-fold more potent at the EP_2_ and DP_1_ receptors, while prostacyclin and beraprost bind to EP_3_ receptors with ~10–20-fold lower affinity [2]. As a consequence of this receptor heterogeneity, selexipag, a highly selective IP receptor agonist with a structure not based on prostacyclin, was developed and now approved for the treatment of PAH [9]. Whilst not a prostacyclin analogue, selexipag is designed as an effective IP receptor agonist (K_i_ for the active metabolite is 20 nM) and for the purpose of this study is considered, along with prostacyclin analogues, as a prostacyclin mimetic. The different activity profiles, drug formulations and administration routes of the collective prostacyclin mimetics (PMs; including epoprostenol sodium, iloprost, treprostinil, beraprost sodium and selexipag), could potentially translate into different clinical responses with varying tolerability. Typical side effects of prostacyclin mimetics include headache, jaw pain, nausea, diarrhoea, and in the case of subcutaneous formulations, site pain is common [9,10,11]. In general patients that tolerate higher doses derive most benefit; however, in a number of patients, the maximum tolerated dose is well below the optimal treatment dose [11]. To overcome dose limitations and the risks associated with continuous ambulatory intravenous therapy, other routes of administration and agents (such as selective IP agonists) have been used.

The relative clinical efficacy and tolerability of PMs remains disputed. Selexipag and its active metabolite, ACT-333679 (formally known as MRE-269), has no reported biological activity at other prostanoid receptors [12]. Consequently, it might have been expected to be associated with fewer adverse events (AEs) [13]. When choosing PM therapies for the management of PAH, patients and clinicians need to weigh the risk of adverse events versus the therapeutic benefit.

Although a recent review has qualitatively explored the sources and management of different adverse events [14], the present analysis focuses on the effect quantitatively, using data gathered systematically from published randomised clinical trials. The primary objective of this systematic review and meta-analysis is to examine the safety and tolerability of PMs in regard to their administration route and subtype. Furthermore, to appreciate the clinical benefit in the context of adverse effects, we meta-analysed relevant outcome measures including: 6-minute walk distance (6MWD), Borg score; haemodynamic parameters; discontinuation and fatalities. Understanding the effects of different treatment types on outcomes will enable clinicians to have a better-informed risk to benefit advice when offering PMs to treat patients who suffer from PAH.

## 2. Experimental Section

### 2.1. Literature Search

The PRISMA and PRISMA-P checklists for systematic reviews and meta-analyses were followed [15,16]. Studies were searched for in the online databases: PubMed/Medline; Scopus; EMBASE; and Cochrane library with no year limits. Highwire, Springer link, Sciencedirect and Wiley library online publisher platforms were also searched. The key words used were: iloprost; ilomedine; ventavis; epoprostenol; Flolan; selexipag; Uptravi; beraprost; treprostinil; UT15; UT-15; UT 15; Remodulin; Tyvaso; Orenitram; pulmonary arterial hypertension; human; patient; and pulmonary hypertension. The medical subject heading (MeSH) terms used were “pulmonary hypertension” and “epoprostenol/analogues and derivatives”. When necessary, the randomised clinical trial (RCT) filters were applied. The search was conducted up until January 2019 and none have been published since. No language limits were used. To our knowledge, and to the date of this submission, no other meta-analyses or RCTs on this topic have been released, other than those referenced in this article [17,18].

### 2.2. Inclusion and Exclusion Criteria for Meta-Analysis

All trials in all years, conducted pre- and post-marketing worldwide, involving IP receptor agonists delivered in any form to adults (aged 16 years or older) for the treatment of PAH which complied to the following criteria were considered: (1) randomised controlled trials versus placebo; (2) patients with PAH who had a follow up period > 8 weeks; and (3) patients who were diagnosed as having PAH according to the World Health Organisation (WHO)/ New York Health Association (NYHA) classification system. Individual case studies, open-label studies, trials in healthy volunteers, abstracts, letters to the editor or trials which had been terminated early due to safety concerns were excluded. Only randomised clinical trial studies which used PMs were included, whilst trials investigating other classes of PAH treatments (phosphodiesterase-5 inhibitors (PDE-5i) and endothelin receptor antagonists (ERAs)), without PMs, were excluded.

### 2.3. Study Quality and Data Extraction

Two reviewers (CP and ME) independently extracted data and assessed the quality of the trials. Uncertainty and disagreement were resolved by consulting a third reviewer (KF). The abstracts, and if appropriate, full text of the articles were assessed against the inclusion and criteria. The investigators then extracted data from the selected studies, including: authors; year of publication; aim of the study; sample size; mean age; gender; and study design. Change from baseline outcome data for treatment and control groups were collected including: 6MWD; Borg Score; change in quality of life (QoL) scores; mortality; patients developing right ventricular heart failure; patients requiring lung transplant or rescue therapy; discontinuation due to Adverse events (AEs); haemodynamic measures (i.e., mixed venous saturation, right atrial pressure, heart rate, pulmonary arterial pressure, pulmonary vascular resistance and cardiac index. AEs, such as site pain, flushing, jaw pain, headache, pain in extremities, diarrhoea, vomiting, arthralgia, nausea, peripheral oedema, fatigue, cough, upper respiratory tract infection (URTI), and dyspnoea were also reported. The quality of the studies (risk of bias) was assessed with the Cochrane Collaboration’s tool [19]. For the meta-analysis, studies were examined for *p*-values, means, and standard deviations or other metrics depicting the effect of PMs on adverse events in PAH. Doses of PMs were analysed by multiplying the mean maximum dose at the end of trial with the dosing regimen (per administration and per day). As the studies were multinational, the international average adult weight was used to calculate dose per day for parenteral routes of administration. To account for differences in bioavailability, the daily doses were multiplied by the reported bioavailabilities for each drug and administration route. For comparison of trial daily dose, two further corrections were made with respect to molecular weight and relative K_i_ (potency) at the IP receptor (further details in supplementary information).

### 2.4. End Points

End point measures included 6MWD, Borg score, discontinuation of treatment, QoL scores and adverse events. Discontinuation refers to patients who did not complete the trial due to intolerable adverse events. Rescue therapy describes when patients are advised by medical professionals to abandon the study protocol to receive approved medication for the benefit of the patient’s health. The death of any patient whilst enrolled in a study was recorded as a fatality. Patient-reported QoL scores were measured by EuroQoL or Minnesota Living with Heart Failure (MLHF) Questionnaires. The EuroQoL visual analogue scale is a self-rated position along a scale between “best and worst imaginable health state” [20]. The MLHF questionnaire is a 21-item assessment of physical activity, subjective symptoms, and psychosocial issues [21]. Reports of AE frequency experienced during the trial period was collected. Although site pain was only applicable to parenteral administration formulations, it was included in this analysis, owing to its reported frequency and impact. No distinction was made between facial and general flushing.

### 2.5. Statistical Analysis

All outcomes were synthesized with a random effects model [22] which was chosen to account for differences in the treatment effects from study to study [23]. The difference of continuous variable or dichotomous data between two groups was estimated by weighted mean difference (WMD) with a two-tailed 95% confidence interval (CI) or an odds ratio (OR) with two-tailed 95% CI, based on the Mantel–Haenszel method [24,25]. Standardised mean difference (SMD) was also used as a summary statistic for continuous outcomes. The SMD values of 0.2, 0.5, and 0.8 were defined as small, moderate, and large effect size, respectively [26,27]. Statistical heterogeneity was assessed using Cochran’s *Q*-test, which examines the null hypothesis that all studies are evaluating the same effect [28]. Statistical significance for heterogeneity was set as *p* ≤ 0.10. Statistic value *I*^2^ was used to quantify the degree of heterogeneity with a score of 25, 50, and 75% representing low, moderate and high levels of inconsistency, respectively [29]. Heterogeneity was further investigated with subgroup analysis and meta-regression. Publication bias was assessed using funnel plots and Egger’s and Begg’s test [30]. *p* < 0.05 was regarded as statistically significant for the outcomes. RevMan software package (Review Manager, Version 5.2, The Cochrane Collaboration, Oxford, UK) and Stata 12.0 (College Station, TX, USA) were employed for statistical analyses.

Subgroup analyses were performed comparing different drug types and different routes of administration. To investigate the effect of therapies given in the 30 days preceding trial initiation, the trials were split into three groups: those given non-PAH specific therapy including oxygen, digoxin, calcium channel blockers, anti-coagulants and diuretics, termed supportive therapy; those given non-prostanoid PAH-specific therapy including endothelin receptor antagonists (ERAs) and phosphodiesterase type 5 inhibitors (PDE-5i); those given prostacyclin therapy which in this case included only epoprostenol. Investigating the effect of background treatment meant dividing trials into two groups: those who were receiving other PAH-specific treatment at a stable monitored dose and those trials in which patients were not. In this case, concomitant therapies included ERA and PDE-5is only. The other groups were defined as not given any PAH-specific therapy on any specific dosing regimen but were treated with supportive therapies (as previously defined) when necessary.

## 3. Results

### 3.1. Study Characteristics

Initial searching highlighted 1802 articles, of which 297 met the RCT filter and search criteria (See Figure S1). Abstract reviewing of the latter identified 35 papers as highly relevant, out of which 14 papers were included in this study. All studies included were multi-centre trials, with a median trial length of 12 weeks (range: 8 to 156). Patients were given PMs via continuous subcutaneous (SC) infusion (treprostinil), continuous intravenous (IV) infusion (treprostinil), repeated daily inhalation (treprostinil, iloprost) or daily oral administration (beraprost, treprostinil, selexipag). Although the quality of assessment of the analysed papers was high, a potential conflict of interest could not be excluded due to funding sources (See Figures S2 and S3).

### 3.2. Patient Characteristics

Within the studies, a total of 3518 patients were included in the meta-analysis; 1846 treated with PMs and 1672 given placebo. Patients enrolled were mostly female (77%) and of a similar age (mean = 47 years, SD = 7) and were diagnosed with mostly Class II (25%) or class III (69%) PAH. The aetiology of PAH patients was mainly idiopathic PAH (68%), with over half of the remaining patients (19%) having connective tissue diseases (CTDs; including scleroderma). Within individual trials, patient cohorts were adjusted for age, gender, and disease severity between placebo and treatment groups. In all trials, patients were receiving non-specific therapy, including seven in which patients were also receiving PAH-specific treatment in the form of an ERA and/or a PDE-5i, described as combination therapy. Where available, the clinical trial report was referred to, including associated unpublished information. A brief description of the trials basic characteristics is shown in Table 1.

### 3.3. Adverse Events

#### 3.3.1. Overall, Drug Type and Route of Administration

Nine adverse events were significantly increased compared to placebo: site pain, headache, jaw pain, nausea, diarrhoea, vomiting, flushing, pain in extremity and arthralgia. Site pain, jaw pain, flushing and headache demonstrated the highest ORs associated with PM therapies (OR 8.7, 4.8, 4.0, 3.6, respectively), although heterogeneity was present, ranging from 0 to 82%.

**Figure 1:**
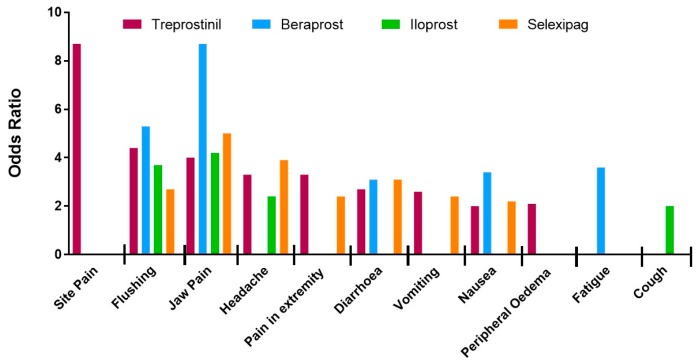
Likelihood of developing each adverse event during a trial period associated with prostacyclin mimetic (treprostinil, beraprost, iloprost and selexipag) treatment as determined by odds ratio.

**Figure 2:**
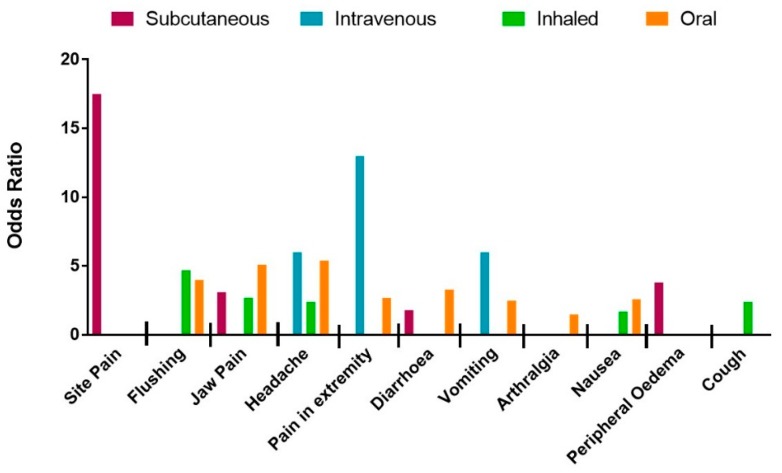
Likelihood, measured by odds ratio, of experiencing each adverse event during trial period associated with prostacyclin mimetic therapy as analysed by route of administration (subcutaneous infusion, intravenous infusion, inhaled therapy and oral tablet.

Subgroup analysis was performed with respect to PM drug type and administration route (Figure 1 and Figure 2, and raw data, see Tables S2 and S3). In brief, the likelihood of site pain was only increased in patients receiving parenteral (IV and SC) treprostinil versus placebo [31,32,33,34], (OR = 8.7; 95% CI: 1.6, 45.9; *I*^2^ = 79%). Furthermore, of the parenteral routes, site pain was most associated with the subcutaneous route of administration (OR = 17.5; 95% CI: 11.1, 27.1; *I*^2^ = 0%). Thirteen studies [31,33,34,36,37,38,39,40,41,42,43] recorded headache as a common side-effect associated with PM therapies (OR = 3.6; 95% CI: 2.4, 5.4; *I*^2^ = 82%). Although headache was not associated with any particular PM drug type, the strongest association was observed with intravenous [34] treprostinil (OR = 6.0; 95% Cl:1.1, 31.5; *I*^2^ not calculated). Vomiting was reported in eight studies [31,32,33,36,37,38,43] and showed a significant increase in patients given PM treatment versus placebo but again there was significant heterogeneity between studies (OR = 2.5; 95% CI: 1.4, 4.7; *I*^2^ = 79%). Subgroup analysis with regard to vomiting did not reveal any association with PM drug type but was most common with the intravenous route of administration (OR = 6.0; 95% CI: 1.1, 31.5; *I*^2^ not calculated).

The likelihood of diarrhoea was increased in patients as reported in twelve studies [31,33,34,35,36,37,38,39,41,42,43,44] (OR = 2.6; 95% CI: 2.0, 3.4; *I*^2^ = 46%). Analysis by different drug types revealed a decrease in heterogeneity for the selexipag group (OR = 3.1, 95% CI: 2.4, 4.1, *I*^2^ = 0%), indicating that the data in each sub-group is in agreement. Diarrhoea was more likely in patients treated with oral PM therapies (OR = 3.3; 95% CI: 2.7, 4.0; *I*^2^ = 0%). Nausea was associated with thirteen studies [31,33,34,35,36,37,38,39,40,41,42,43,44] with moderate heterogeneity, (OR = 2.2; 95% CI: 1.7, 2.8; *I*^2^ = 41%). When analysed by drug type, the magnitude was greatest for patients taking beraprost (OR = 3.4; 95% CI: 1.7, 6.7; *I*^2^ = 0%). Eleven studies recorded cutaneous flushing as an adverse event [33,35,36,37,38,39,40,41,42,43]. Meta-analysis calculated a strong significant increase in OR with limited heterogeneity (OR = 4.0; 95% CI: 3.0, 5.3; *I*^2^ = 19%). Subgroup analysis showed association with beraprost (OR = 5.3; 95% CI: 2.3, 12.1; *I*^2^ = 49%) and inhaled therapy (OR = 4.7; 95% CI: 2.0, 11.1; *I*^2^ = 26%). The thirteen studies [31,33,34,35,36,37,38,39,40,41,42,43,44] reporting jaw pain showed a significant association with PMs versus placebo (OR = 4.6; 95% CI: 3.6, 5.8; *I*^2^ = 0%). This association was greatest for treatment with beraprost (OR = 8.7; 95% CI: 2.0, 37.2; *I*^2^ = 50%) and oral PMs (OR = 5.1; 95% CI: 4.0, 6.7; *I*^2^ = 0%).

The eight studies [33,34,36,37,38,40,42,43] reporting pain in the extremity showed significant and homogenous association with PM treatment (OR = 2.7; 95% CI: 1.7 3.5; *I*^2^ = 0%). Pain in extremities was mostly associated with intravenous administration of PMs (OR = 13.0; 95% CI: 1.5, 112.3; *I*^2^ not calculated). Arthralgia was reported in four of the studies [36,37,38,43] featuring only oral treprostinil and selexipag, and showed a strong increase in OR in the treatment groups with no heterogeneity (OR = 1.5; 95% CI: 1.1, 2.2; *I*^2^ = 0%). Seven studies [31,37,38,39,40,43,44] reported peripheral oedema although on meta-analysis this was not more frequent with active therapy when compared to placebo (OR = 1.3; 95% CI: 0.8, 2.1; *I*^2^ = 59%). However, on subgroup analysis the likelihood of peripheral oedema was significantly increased for patients treated with treprostinil (OR = 2.1; 95% CI: 1.2, 3.8; *I*^2^ = 18%). Subcutaneous administration, which only included treprostinil treatment, was also associated with an increase in peripheral oedema (OR = 3.8; 95% CI: 1.5, 9.6; *I*^2^ not calculated).

Fatigue was reported in seven trials [35,36,37,38,40,43], although no significant difference in incidence between placebo and treatment groups with significant heterogeneity was detected (OR = 1.2; 95% CI: 0.8, 1.8; *I*^2^ = 50%). When analysed by PM drug type, heterogeneity was eliminated and a significant increase in OR of fatigue in patients treated with beraprost was observed (OR = 3.6; 95% CI: 1.1, 11.8; *I*^2^ not calculated). Seven trials reported cough as a side effect [34,35,37,40,42,43,44], which when meta-analysed, showed no significant difference between treatment and placebo groups (OR = 1.5; 95% CI: 0.9, 2.6 *I*^2^ = 68%). Subgroup analysis revealed a significant increase in the OR of cough for patients in the inhaled PMs (OR = 2.4; 95% CI: 1.6, 3.5; *I*^2^ = 0%) and iloprost groups (OR = 2.0; 95% CI: 1.2, 3.5; *I*^2^ = 0%). Despite showing no significant correlation in the overall meta-analysis, when compared by PM types, the likelihood for upper respiratory tract infection (URTI) was shown to be negatively associated with beraprost treatment (OR = 0.5; 95% CI: 0.2, 1.0; *I*^2^ not calculated). A similar decreased association was found for patients experiencing dyspnoea when treated with selexipag (OR = 0.7; 95% CI: 0.5 to 1.0; *I*^2^ not calculated).

#### 3.3.2. Pre-Trial and Concomitant Therapy

The effect of therapeutic treatments on side-effect profiles, either in the run-up to the trial or taken concomitantly was also investigated, with data shown in Table S4. Headache (OR = 5.2; 95% CI: 3.2, 8.3; *I*^2^ = 69%), vomiting (OR = 3.6; 95% CI: 1.6, 8.2; *I*^2^ = 86%) and diarrhoea (OR = 3.2; 95% CI: 2.5, 4.3; *I*^2^ = 28%) were more prevalent in patients receiving PM therapy in combination with other PAH specific therapies than those on monotherapy. This trend was also seen for trials in which patients received PDE-5 inhibitors and/or ERAs in the 30-day period prior to randomisation. The only AE significantly increased upon transitioning from epoprostenol pre-trial therapy to treprostinil was site pain. No significant difference was shown between placebo and treatment groups for the likelihood of experiencing dizziness, insomnia, abdominal pain, palpitations or chest pain (see Table S5).

#### 3.3.3. Trial Dosing

The mean dose reached by patients recorded for each trial was analysed accounting for the bioavailability, molecular weight and potency, allowing for comparison of dose effects. Greater doses were achieved for intravenous treprostinil (4.77) and one trial using subcutaneous treprostinil (2.87 mg). Similar doses of oral treprostinil (average: 1.10 mg) and selexipag (average: 0.75 mg) doses were achieved. Taking into account molecular weight and differences in potencies at the IP receptor, broadly equivalent doses were achieved in 8/14 clinical trials. The lowest relative doses were seen for the two beraprost trials (average: 7.7-fold lower) (see Table S1).

### 3.4. Efficacy Outcomes

#### 3.4.1. MWD, Borg Score, Haemodynamics and Quality of Life Score

**Figure 3:**
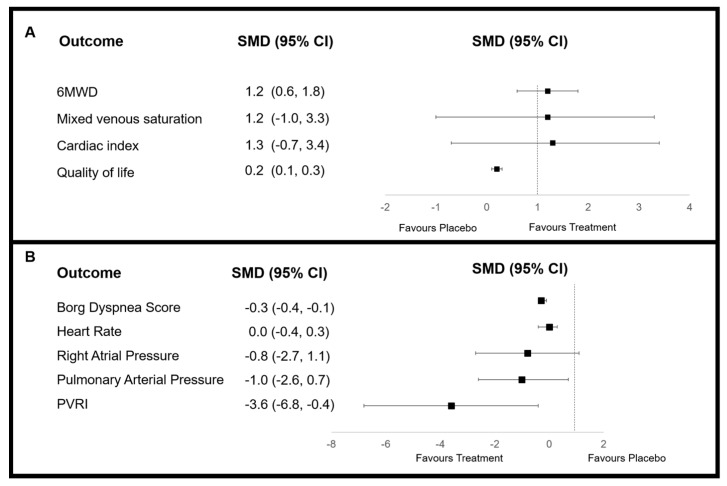
Forest plots of overall change in meta-analysed outcome data for prostacyclin mimetic therapy as determined by standardised mean difference (SMD), where (**A**) an increased value favours treatment and (**B**) a decreased value favours treatment. CI = confidence interval; PVRI = mean change in pulmonary vascular resistance index. Pulmonary arterial pressure refers to mean pressure recorded.

Study interventions were overall effective at increasing 6MWD by 16.3 m (95% CI: 13.0, 19.7; *I*^2^ = 90%). Intravenously administered drugs (treprostinil) were more effective with WMD 93.0 m (95% CI: 8.3, 178.0; *I*^2^ = 0%), in comparison to oral WMD 15.3 m (95% CI: 11.1–19.5; *I*^2^ = 93%). Overall, patient-reported Borg score was decreased by PM therapies to a limited extent (SMD = −0.3; 95% CI: −0.4, −0.1). However, by subgroup analysis, this was significant for beraprost [39,41] (SMD = −0.4; 95% CI: −0.6, −0.1; *I*^2^ = 0%) as well as in trials using intravenous PMs (SMD = −1.0; 95% CI: −1.6, −0.3; *I*^2^ not calculated) or where patients received monotherapy only [31,32,34,35,40] (SMD = −0.4; 95% CI: −0.6, −0.1; *I*^2^ = 32%). Quality of life (QoL) was significantly improved in the inhaled group SMD = 0.3 (95% CI: 0.1, 0.6; *I*^2^ = 9%). In comparison, no significant improvement was detected for the oral group, although QoL was not measured in trials with other routes of administration. Mean change in pulmonary arterial pressure was significantly reduced with PM therapy (SMD = −1.0; 95% CI: −2.6, −0.7; *I*^2^ = 99%). When analysed by subgroups, significance was only observed for the drug type beraprost (SMD = −1.7; 95% CI: −4.1, 0.6; *I*^2^ = 0%), as well as therapies administered by inhalation (SMD = −0.2; 95% CI: −0.8, 0.3; *I*^2^ = 71%) and orally (SMD = −0.3; 95% CI: −0.5, 0.0; *I*^2^ = 0%). Pulmonary vascular resistance index was significantly decreased by PM therapy (SMD = −3.6; 95% CI: −6.8, −0.4; *I*^2^ = 99%). The greatest decrease was seen for the subcutaneous subgroup (SMD = −5.5; 95% CI: −10.1, −0.9; *I*^2^ = 98%), although heterogeneity was high. Heterogeneity was, however, largely reduced for the beraprost subgroup (SMD = −1.8; 95% CI: −2.2, −1.5; *I*^2^ = 5%). Mixed venous saturation improvements were shown for inhalation therapies [31,32,39,42,44] (WMD = 0.3; 95% CI: 0.0, 0.6; *I*^2^ not calculated) (data presented in Figure 3 and raw data provided in Tables S6–S9).

#### 3.4.2. Fatalities, Discontinuation from Adverse Event

**Figure 4:**
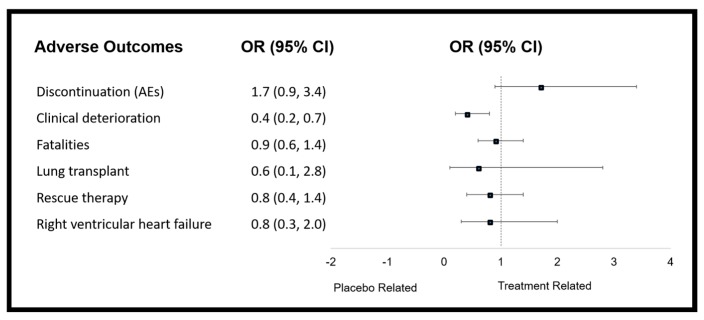
Forest plot showing the overall changes in efficacy measures of prostacyclin mimetic therapy as determined by odds ratio.

Discontinuations due to adverse events were monitored in 10 trials, and occurred in 9 trials [32,33,34,36,37,41,43,44]. In comparison to placebo, patients were significantly more likely to discontinue PMs in trials involving those orally administered [30,31,32,34,35,36,37,41,43] (OR = 2.1; 95% CI: 1.5, 3.0; *I*^2^ = 0%). This included, beraprost, oral treprostinil and selexipag. An increased trend was seen for all three of the oral PM trials analysed, although when considering PM type, this reached significance for only selexipag (OR = 2.2; 95% CI: 1.5, 3.3; *I*^2^ not calculated). Mortality was reported in 11 studies but OR could not be calculated for two, in which no deaths were reported. Therefore, nine studies were included in the analysis. Although the OR of PM subgroups in comparison to placebo were not statistically significant in relation to mortality, the intravenous and inhaled PMs group, including treprostinil and iloprost, showed a strong trend in reducing mortality. Analysed by separating into PM type, there was no change in the likelihood of mortality, acquirement of lung transplant, rescue therapy and the development of right ventricular heart failure (Figure 4 and data shown in Tables S10 and S11).

#### 3.4.3. Publication Bias and Heterogeneity Analysis

On testing funnel plot symmetry and the Beggs and Eggers test, there was no publication bias detected for: 6MWD (SMD and WMD); Borg score (SMD and WMD); mixed venous saturation (SMD and WMD); right atrial pressure (SMD and WMD); cardiac index (SMD and WMD); heart rate (SMD and WMD); mortality; rescue therapy; and discontinuation due to the adverse events. Heterogeneity was not attributed to differences in the ratios of patients with class III IPAH and gender, nor was there a difference in the mean age for 6MWD (SMD and WMD), Borg score (WMD), mortality, rescue therapy, and discontinuation due to the adverse events (see Tables S12 and S13).

## 4. Discussion

In patients with PAH, this meta-analysis investigated the effects of PMs, and their route of administration on the likelihood of potential AEs. Here, it was demonstrated that the AEs continued to burden PAH patients in all treatments targeting the IP receptor. Altering the route of administration and the receptor selectivity of PMs may change the AE profile, but it did not appear to significantly reduce or prevent their incidence. Diarrhoea was associated with the enteral route of administration but was not alleviated by pro-drug formulation. Vomiting and headache were associated with intravenous PM administration, but not with subcutaneous. An improved QoL was significant for patients receiving inhaled PMs.

### 4.1. Main Findings

Adverse events including: site pain; headache; jaw pain; nausea; vomiting; diarrhoea; cough; peripheral oedema; and arthralgia were significantly increased in the PM treatment group in comparison to placebo. Treprostinil displayed the greatest number of adverse events and site pain was responsible for the highest likelihood of any adverse event. The latter was increased nine-fold in the treatment group in comparison to placebo. This finding is consistent with other published literature [45] and is in line with clinical reports [46]. Selexipag was associated with the highest likelihood of headache. Only inhaled iloprost increased likelihood of cough, and despite previous reports, showed no association with gastrointestinal AEs [47]. Beraprost was associated with the least types of AEs, though flushing, jaw pain and nausea were most common with this drug.

Although the central causes of nausea and vomiting cannot be underestimated [48], the increased likelihood of nausea associated with oral route of administration may be attributed to local vagal stimulation. Gastrointestinal (GI) AEs may be reduced by drug administration strategies. For example, selexipag is administered in its inactive pro-drug form. After absorption and conversion to its more active metabolite, ACT-333679, it can activate IP receptors with a 10-fold higher potency, than the pro-drug [12,49]. The pro-drug form is expected to achieve steady blood concentrations and reduce GI AEs [13], which may explain the lower likelihood of nausea in patients taking selexipag. However, the pro-drug formulation did not reduce the likelihood of vomiting or diarrhoea, suggesting these AEs are driven by a systemic mechanism. Clinically, the relaxation of non-diseased systemic blood vessels by PMs is known to result in hypotension at high concentrations and is associated with side effects such as dizziness, nausea, headache and palpitations [50,51]. Of the three studies that reported systemic blood pressure (SBP) measurement, none showed a significant difference between SBP in patients taking drugs and placebo. It cannot, however, be ruled out that nausea and headache is attributable to a reduction in SBP in the other studies where SBP was not reported.

Cutaneous flushing was increased in patients taking inhaled and oral PM therapy. Compounds such as nicotinic acid are known to cause flushing by activating the DP_1_ receptor, which is found in abundance in the skin [52,53,54]. The promiscuous activity of some PMs across different prostanoid receptors, makes it possible for the flushing effect experienced in PAH therapies to be DP_1_ receptor driven. Consistent with this hypothesis, flushing effects were prevalent for the treprostinil group, which has the most potent DP_1_ activity (K_i_ 4.4 nM), but less prevalence for the selexipag group, which has no reported DP_1_ activity [2]. Conversely, oral beraprost was also highly associated with flushing, despite having little potency at the DP_1_ receptor (K_i_ > 10 µM) [12]. The association of flushing with beraprost and the selective IP receptor, selexipag, may suggest that the IP receptors drive the effect but might combine with the DP_1_ receptor to produce a greater effect. Upon inhalation of the PMs and subsequent exhalations, PM may come into contact with the skin surrounding the mouth, leading to a flushing effect. This may explain facial flushing but no flushing at other sites of body, particularly the torso. The low association of flushing with parenteral administration of treprostinil, which achieves near-constant blood concentrations, could also suggest flushing to be associated with DP_1_ and/or IP receptor agonism in response to fluctuations of the drug in the blood, or might indicate receptor desensitisation.

Treprostinil is the only drug to be administered subcutaneously and intravenously, and hence, was the only PM to be associated with site pain. The injection site pain was associated with subcutaneous infusion. Additionally, treprostinil was the only PM to show a significant increase in peripheral oedema, but this was only observed when the drug was administered subcutaneously. Site pain may be a consequence of the direct interaction of treprostinil with vasculature in the skin which is rich in inflammatory mediating receptors IP, DP_1_, EP_2_, and EP_4_ [53,55], and to which treprostinil will bind with high affinity to the first three but low affinity for EP_4_ [2]. Whether this is a unique side-effect to treprostinil is unknown, although it could be further investigated with subcutaneous administration of other PMs. The strong trend of site pain seen in the clinical setting when treprostinil is administered subcutaneously, as well as the inconvenience of pump management, has driven on-going trials into pro-drug formulations [56] and implantable pumps [57] for treprostinil that may reduce site pain. There is considerable interest and clinical need for upfront combination therapies and clinical evidence suggests a clear benefit to combination PAH therapy [9,58,59]. This meta-analysis shows ERAs and PDE-5i given alongside PMs were associated with increased headache and vomiting. The administration of one or more anti-hypertensive agents can lead to increased cranial blood flow and intracranial pressure [60,61], which may explain the increased incidence of headache with patients on a combination of therapies that are all designed to promote vasodilation. Furthermore, increases in intracranial pressure can be associated with vomiting [62]. Strategies to avoid the AEs discussed are needed to gain optimal clinical benefit.

Patient experience was interpreted using patient reported outcomes. The decrease in Borg scores suggest patients experienced symptomatic relief overall during the trials. However, this benefit was significant only for beraprost when analysed by drug type. When investigating Borg score improvements by administration route, orally administered PMs were associated with a similarly modest improvement, though the most significant change was seen for PMs administered intravenously. Quality of Life scores were measured using the MLHF Questionnaire or the EuroQol visual-analogue scale. Although, there was an improvement in QoL with the inhaled therapies, this was not the case with oral PMs. Moreover, the limited changes in QoL scores indicate that the benefits associated with more favourable treatment options are still hindered by treatment impact on day-to-day living. Our results confirm that inhalation therapy is viewed as the administration route with the most improved quality of life, despite the frequent administration regime.

Discontinuation of treatment due to AEs is an indicator of tolerability. Our study found no overall significant increase in likelihood of discontinuation with PM therapies. However, selexipag was associated with a tendency for discontinuations, reported to be caused by headache, diarrhoea and nausea in the trials [43]. When analysed by administration route, oral PMs also showed an increased likelihood of discontinuation. The selexipag trial period was greater than for the other trials so the association between selexipag and discontinuations result should be considered in respect of significant discontinuations owing to adverse events for subcutaneous treprostinil in a similar extended study period [63]. Although a large variation in treatment response and tolerability in PAH patients is observed clinically, it is well understood that patients who tolerate greater drug doses derive the greatest clinical benefit. Patients are advised to up-titrate treatment to the maximum tolerated dose, which for many patients is well below the optimal treatment dose [64,65]. Thus, clinicians should focus on mitigating dose-limiting adverse events in the first instance to allow for higher doses to be administered. When clinical benefits are observed, adverse events such as nausea, headache and site pain can be treated respectively with anti-emetics, systemic analgesics and topical analgesics. Circumventing additional AEs which do not limit dose but contribute to a lower quality of life for the patient, can be treated in a similar manner and so contribute to increased tolerance in the long-term. Monitoring the patient experience using QoL scores can help engagement of treatment progression between patient and clinician which can help to best tailor treatment to each patient. Dose of PM was expected to have some influence on effect outcome efficacy as well as the likelihood of adverse events. Considering differences in bioavailability, molecular weight and IP receptor potency, parenteral administration achieves greater doses of PM. The doses of selexipag and oral treprostinil appeared to be pharmacologically similar, but in comparison to all other trials with oral PMs, oral beraprost may have been under-dosed.

Prostacyclin mimetic therapy is effective in improving patients 6MWD in comparison to placebo controls. This effect was greater for IV, consistent with a previous meta-analysis [66]. Six-minute walk distance was reported for all the studies which showed an overall improved 6WMD of 16.3 m, less than previously calculated (range: 33 to 41.8) [67,68], and less than the reported minimal important distance (33 m) [69] or distance associated with clinical benefit (41.8 m) [70]. In some instances, modest improvement may be a consequence of under dosing. For example, in the FREEDOM-C study, a 34-m and 18-m improvement in 6MWD was reported in those patients receiving 16 mg and 3.25 mg treprostinil, respectively, but only 4 m with doses < 1 mg or when the drug was discontinued because of adverse events [36]. Overall, the pulmonary vascular resistance index was shown to be significantly decreased in patients taking PMs, including for the following subgroups (administrations: subcutaneous, oral; and PM type: treprostinil, beraprost).

### 4.2. Strengths and Limitations

Previous meta-analyses have included all types of PAH treatment or focused on survival and outcomes [17,18,66,68]. To our knowledge, this is the first study to quantify the likelihood of adverse events associated with PM therapy in PAH patients, analysing via meta-analysis in blinded, randomised, placebo-controlled trials. The novelty of this study lies in the comparative investigation of the different PMs and their route of administrations on AEs, thus allowing for AE-mediating mechanisms to be elucidated. The inclusion of selexipag permitted investigation into the effect of a selective IP receptor agonist and an oral pro-drug on AE likelihood. It has previously been suggested that such a structure would give rise to fewer and less intense AEs [64] or a better tolerability compared to prostanoid therapies [11], although our quantitative analysis does not support such an interpretation. We collected data from 14 blinded, placebo-controlled, randomised clinical trials and analysed their reported results, which included 3518 patients, where 50% of the trials had ≥100 patients in both the treatment and placebo group. The inclusion of additional trials involving epoprostenol treatment would have been an interesting comparison group; unfortunately these studies were unblinded and did not report adverse event data that could be analysed [71,72]. The quality of the trials overall was acceptable, and data was collected to the best of our abilities from trial reports. The aim was to focus on the patient experience of PM therapy, so the approach included data on AEs reported, the patient-reported outcome, Borg score and QoL scores. Nonetheless, our analysis is not without limitations; most studies were funded by the drug manufacturer, therefore reporting and publication bias cannot be excluded. Odds ratio was used to report likelihood of events which does not take trial duration into consideration [59]. Inconsistent reporting of 6MWD and Borg score (mean versus median, SD versus SE) was overcome by converting medians to means and interquartile ranges to SE with standard formulae. The Barst study for beraprost was a 12-month trial and although outcome data could be obtained after 12 weeks, AEs were not [39]. Data obtained for adverse event analysis were cumulative data over a 12-month period. Selexipag data from the GRIPHON trial were also obtained after 26 weeks for outcome data but after the end of the trial for adverse event data [43]. This may exaggerate odds ratios for these trials. However, trials also found that most events occurred in the titration period at the beginning of trials, so we believe the trials to still be comparable [43]. Direct correlation of AE profiles and PM type was hindered by drugs with multiple administration routes. For one study [43], discontinued patient data were reported as a zero change in 6MWD, which potentially hides negative effects. Owing to the relatively small number of studies included and heterogeneity between trial design, our study conclusions may be limited. The interesting finding that the selective IP receptor agonist, selexipag, has a similar side effect profile to other analogues warrants further investigation. This is relevant to the clinician and the patient when considering treatment options.

## 5. Conclusions

This investigation provides a summary of efficacy and adverse event profiles associated with PM therapy for the treatment of PAH. It aims to help patients and clinicians in their decision to weigh the risks and benefits of PAH therapies. All treatments were effective at improving exercise proficiency, although none avoided the burden of AEs. Subgroup analysis was used to determine trends in reported AEs, and possible mechanisms for their exacerbation were discussed. While selexipag had the most significant discontinuation rate, treprostinil caused significant site pain and beraprost had double the likelihood of jaw pain. Delivery of PMs by inhalation has shown superiority in improving QoL when compared to orally administered PMs. Importantly, the majority of AEs were likely to be mediated by the IP receptor pathway, given that the selective IP receptor agonist (selexipag) demonstrated a similar AE profile to other non-pro-drug oral PMs. Effective and tolerable PAH therapies should aim to decrease exposure of PMs to prostanoid receptors located in non-target sites, particularly the skin.

## Figures and Tables

**Table 1:** Summary of clinical trials involving prostacyclin mimetics compared against placebo.

Study	Drug	Admin. Route	Study Length/Weeks	Mean Daily Dose/mg ^#^	Therapy Type	Pre-Trial Therapy	Treatment Patients	Control Patients	% NYHA Class III	% IPAH	% CTD	% Female	Mean Age/year
Simonneau et al., 2002 [31]	Treprostinil	SC	12	0.83 **^#^**	Monotherapy	30 days no prostanoids	233	236	81	58	17	81	44.5
McLaughlin et al., 2003 [32]	Treprostinil	SC	8	1.16 **^#^**	Monotherapy	Supportive therapy *	15	9	-	100	0	-	-
Rubenfire et al., 2007 [33]	Treprostinil	SC	8	2.87 **^#^**	Monotherapy	Patients must have been receiving epoprostenol therapy for 3 months	14	8	41	71	14	86	45.5
Hiremath et al., 2010 [34]	Treprostinil	IV	12	4.77 **^#^**	Monotherapy	Supportive therapy *	30	14	95	95	5	61	32
McLaughlin et al., 2010 [35]	Treprostinil	Inhaled	12	1.40	Combination (Bosentan)	Bosentan for 3 months	115	120	98	56	35	81	54
Tapson et al., 2012 [36]	Treprostinil	Oral	16		Combination (ERA and/or PDE-5i)	PDE-5i and or ERA for +3 months at a stable dose for 1 month, no prostanoids	174	176	74	65	28	82	51
Jing et al., 2013 [37]	Treprostinil	Oral	12	1.16	Monotherapy	Supportive therapy *	233	116	66	73	19	73	39.4
Tapson et al., 2013 [38]	Treprostinil	Oral	16	1.05	Combination (ERA and/or PDE-5i)	PDE-5 inhibitor and or ERA for +3 months at a stable dose for 1 month, no prostanoids	157	153	73	66	31	78	54
Barst et al., 2003 [39]	Beraprost	Oral	52	0.17	Monotherapy	Supportive therapy *	60	56	47.4	78	10	85.3	42
McLaughlin et al., 2006 [40]	Iloprost	Inhaled	12	0.12	Combination (Bosentan)	Bosentan for 4 months	34	33	94	55	45 ^∞^	79	50
Galie et. Al. 2002 [41]	Beraprost	Oral	12	0.19	Monotherapy	Not prostanoid treatment for 1 month	65	65	50.8	54	8	61.5	45.5
Simonneau et al., 2012 [42]	Selexipag	Oral	17	0.57	Combination (ERA and/or PDE-5i)	Stable dose ERA and PDE-5i for 12 weeks	33	10	60.5	72	12	81.4	54.6
Sitbon et al., 2015 [43]	Selexipag	Oral	156	0.93	Combination (ERA and/or PDE-5i)	Stable dose ERA and PDE-5i for 12 weeks	582	574	52.5	56	29	79.8	48.1
Olschewski et al., 2002 [44]	Iloprost	Inhaled	12	0.03	Monotherapy	Supportive therapy *	101	102	58.6	50	12	67.5	52

NYHA = New York Heart Association; PAH= pulmonary arterial hypertension; IPAH = idiopathic PAH; CTD = connective tissue disease; SC = subcutaneous; IV = intravenous; ERA = endothelin receptor antagonist; PDE-5i = phosphodiesterase-5 inhibitor. * Includes non-PAH specific therapy including oxygen, digoxin, calcium channel blockers, anti-coagulants and diuretics. ^#^ See experimental section for details. ^∞^ Disease-associated-PAH included CTD but not exclusively.

## Supplementary Materials

The following are available online at https://www.mdpi.com/2077-0383/8/4/481/s1, Dose analysis methods, Figure S1: Literature search results flowchart, Figure S2: Assessment of bias, Figure S3: Assessment of study quality, Table S1: Table of doses; Table S2: Table of adverse events as meta-analysed by drug type, Table S3: Odds ratios of adverse events as meta-analysed by route of administration; Table S4: Odds ratio of adverse events as meta-analysed by the treatment received in the one month up to the trial date; Table S5: Odds ratios of adverse events as meta-analysed by the concomitant therapy given during the length of the trial; Table S6: Weighted mean difference of outcomes as meta-analysed by drug type; Table S7: Standardised mean difference of outcomes as meta-analysed by drug type; Table S8: Standardised mean difference of outcomes as meta-analysed by route of administration; Table S9: Weighted mean difference of outcomes as meta-analysed by route of administration, Table S10: Odds ratios of outcomes as meta-analysed by drug type; Table S11: Odds ratios of outcomes as meta-analysed by route of administration; Table S12: Meta-regression for outcomes; Table S13: Eggers and Beggs values tests detecting publication bias on overall groups.
