# Supplementary material for: Adverse Events of Prostacyclin Mimetics in Pulmonary Arterial Hypertension: A Systematic Review and Meta-Analysis
Source: J Clin Med. 2019 Apr 9;8(4):481. doi: 10.3390/jcm8040481 (PMC6517977; doi:10.3390/jcm8040481)
Supplement: Supplementary file 1 [file jcm-08-00481-s001.pdf]

Dose Analysis Methods

Doses were analysed by multiplying the mean maximum dose at the end of trial with the dosing regime (per administration, per day). As studies were multinational, the international average adult weight was used to calculate dose per day for parenteral routes of administration. To account for differences in bioavailability, the daily doses were multiplied by the reported bioavailabilities for each drug and administration route (SI table 1). To assess pharmacological equivalence, drug doses were normalized to the iloprost dose reported in the McLaughlin et al. 2006 clinical trial and given 1 for both being the lowest molecular weight and the highest Ki at the IP receptor (4 nM). The estimated dose-ratio or all PMs with respect to iloprost is given in the last column

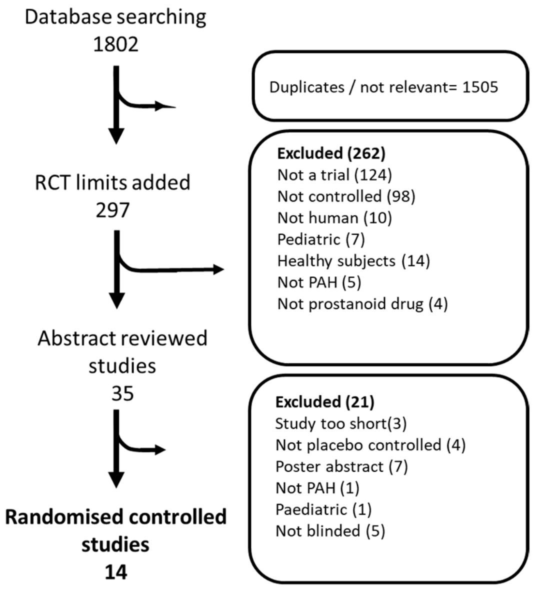

Figure S1. Literature search results flowchart.

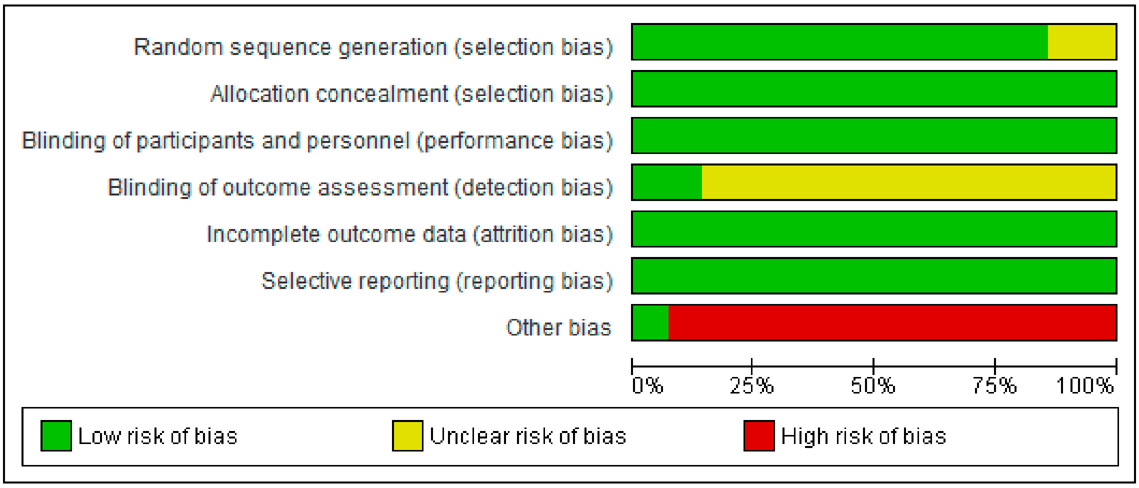

Figure S2. Assessment of bias.

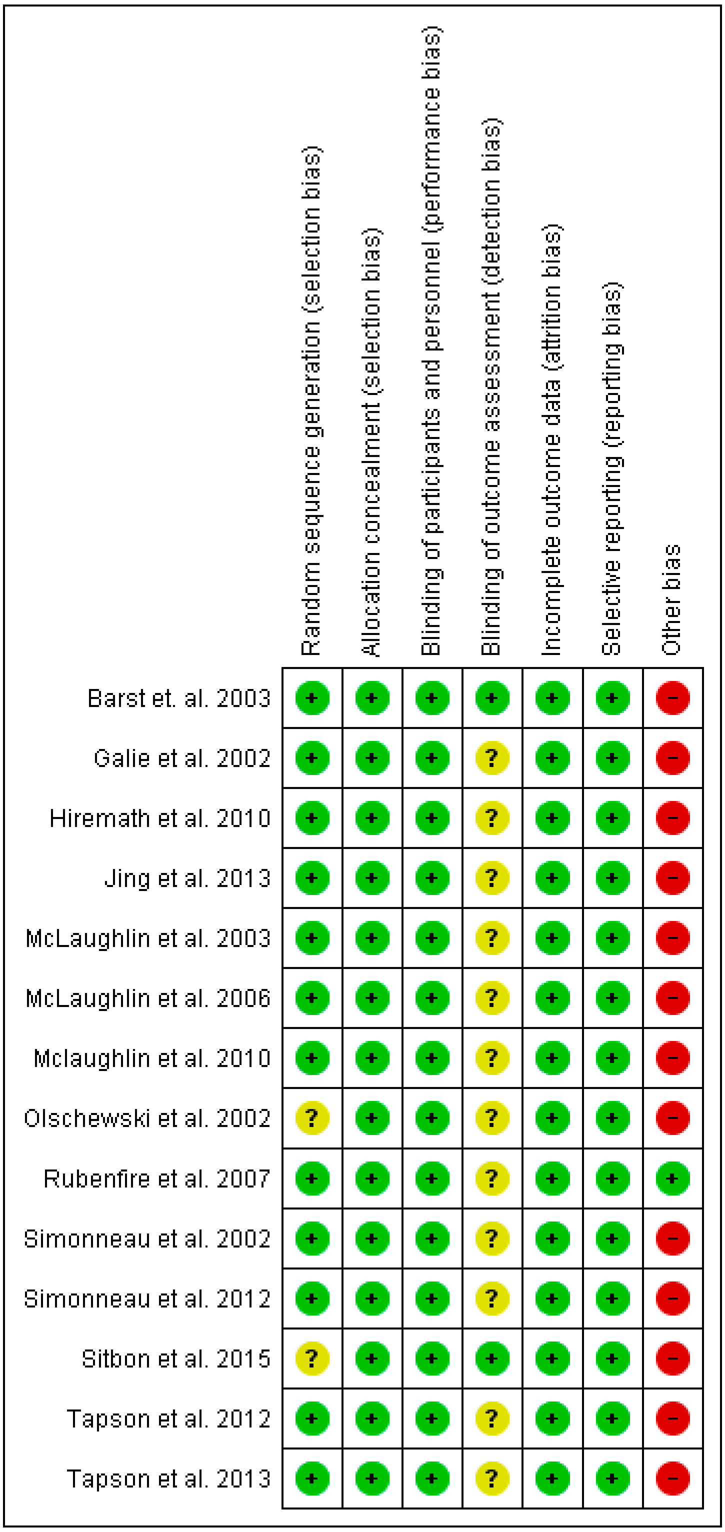

Figure S3. Assessment of study quality.

Table S1. Dosing information for each trial.

Trial	Administration Route	PM	Dose per Administration	Daily Dosing Regime	Bio-Availability [ref]	Daily Dose (mg)	Relative Dose Corrected for IP Potency /mg
Simonneau et al., 2002	Subcutaneous	Treprostinil	9.3 ng/kg/min	Continuous	1 [1]	0.830 †	0.76
McLaughlin et al., 2003	Subcutaneous	Treprostinil	13 ng/kg/min	Continuous	1 [1]	1.161 †	1.06
Rubenfire et al., 2007	Subcutaneous	Treprostinil	32.2 ng/kg/min	Continuous	1 [1]	2.875 †	2.62
Hiremath et al., 2010	Intravenous	Treprostinil	72 ng/kg/min	Continuous	1 [1]	4.769 †	4.34
McLaughlin et al., 2010	Inhaled	Treprostinil	50 µg	9 inhalations QD	0.78 [2]	1.404	1.28
Tapson et al., 2012	Oral	Treprostinil	-	-	-	-	-
Jing et al., 2013	Oral	Treprostinil	3.4 mg	BD	0.17 [3]	1.156	1.05
Tapson et al., 2013	Oral	Treprostinil	3.1 mg	BD	0.17 [3]	1.054	0.96
Simonneau et al., 2012	Oral	Selexipag	583 µg	BD	0.49 [4]	0.571	0.69
Sitbon et al., 2015	Oral	Selexipag	950.4 µg	BD	0.49 [4]	0.931	1.13
Galie et al., 2002	Oral	Beraprost	80 µg	QD	0.6 [5]	0.192	0.14
Barst et al., 2003	Oral	Beraprost	71 µg	QD	0.6 [5]	0.170	0.12
McLaughlin et al., 2006	Inhaled	Iloprost	26.8 µg	5.6 inhalations *	0.8 [6]	0.120	1.00
Olschewski et al., 2002	Inhaled	Iloprost	4.8 µg	7.5 inhalations *	0.8 [6]	0.029	0.24

PM = prostacyclin mimetic; BD = two dosings daily; QD = four dosing; * = Mean number of inhalations achieved during trial; † = weight based on international average (62 kg). Daily dose is calculated as described in the methods, accounting for mean dose of administration, number of dosings per day, bioavailability, molecular weight and potency at the IP receptor. Mw treprostinil, selexipag, beraprost and iloprost = 412.5, 496.6, 420.5, 360.5 g/mol, respectively and for potency, Ki 4, 20, 32,38 nM, respectively.

Table S2. Table of adverse events (AE) as meta-analysed by drug type.

AE	Overall OR (CI)<br>I^2, p Value	Treprostinil OR (CI)<br>I^2, p Value	Beraprost OR (CI)<br>I^2, p Value	Iloprost OR (CI)<br>I^2, p Value	Selexipag OR (CI)<br>I^2, p Value
Site pain	**8.7**<br>(1.6, 45.9)<br>79%, 0.003	**8.7**<br>(1.6, 45.9)<br>79%, 0.003	-	-	-
Flushing	**4.0**<br>(3.0, 5.3)<br>19%, 0.259	**4.4**<br>(2.7, 7.1)<br>30%, 0.02	**5.3**<br>(2.3, 12.1)<br>49%, 0.16	**3.7**<br>(1.8, 7.4)<br>0%, 0.886	**2.7**<br>(1.7, 4.1)<br>0%, 0.675
Jaw Pain	**4.6**<br>(3.6, 5.8)<br>0%, 0.571	**4.0**<br>(2.9, 5.6)<br>0%, 0.421	**8.7**<br>(2.0, 37.2)<br>50%, 0.159	**4.2**<br>(1.6, 10.8)<br>0%, 0.885	**5.0**<br>(3.4, 7.3)<br>0% 0.356
Headache	**3.6**<br>(2.4, 5.4)<br>82%, <0.001	**3.3**<br>(1.6, 6.8)<br>88%, <0.001	NS<br>87%, 0.006	**2.4**<br>(1.0, 5.7)<br>49%, 0.161	**3.9**<br>(3.0, 5.0)<br>0%, 0.406
Pain in extremity	**2.7**<br>(1.7, 3.5)<br>0%, 0.480	**3.3**<br>(2.2, 4.9)<br>0%, 0.421	-	NS<br>- , -	**2.4**<br>(1.7, 3.5)<br>0%, 0.354
Diarrhoea	**2.6**<br>(2.0, 3.4)<br>46%, 0.039	**2.7**<br>(1.9, 3.9)<br>47%, 0.079	**3.1**<br>(1.4, 7.3)<br>36%, 0.213	NS<br>- , -	**3.1**<br>(2.4, 4.1)<br>0%, 0.698
Vomiting	**2.5**<br>(1.4, 4.7)<br>79%, <0.00	**2.6**<br>(1.1, 6.1)<br>79%, <0.001	-	-	**2.4**<br>(1.7, 3.4)<br>- , -
Arthralgia	**1.5**<br>(1.1, 2.2)<br>0%, 0.788	NS<br>0%, 0.628	-	-	NS<br>- , -
Nausea	**2.2**<br>(1.7, 2.8)<br>41%, 0.060	**2.0**<br>(1.3, 3.0)<br>63%, 0.012	**3.4**<br>(1.7, 6.7)<br>0%, 0.451	NS<br>0%, 0.588	**2.2**<br>(1.7, 2.9)<br>0%, 0.386
Peripheral Oedema	NS<br>59%, 0.024	**2.1**<br>(1.2, 3.8)<br>18%, 0.297	NS<br>- , -	NS<br>0%, 0.889	NS<br>- , -
Fatigue	NS<br>50%, 0.062	NS<br>0%, 0.726	**3.6**<br>(1.1, 11.8)<br>- , -	NS<br>- , -	NS<br>- , -
Cough	NS<br>68%, 0.005	NS<br>68%, 0.042	-	**2.0**<br>(1.2, 3.5)<br>0%, 0.042	NS<br>0%, 0.376
URTI	NS<br>6%, 0.382	NS<br>0%, 0.486	**0.5**<br>(0.2, 1.0)<br>- , -	NS<br>- , -	NS<br>0%, 0.726
Dyspnea	NS<br>57%, 0.030	NS<br>59%, 0.046	-	NS<br>- , -	**0.7**<br>(0.5, 1.0)<br>- , -

OR = odds ratio; CI = confidence interval; UTRI = upper respiratory tract infection; NS = Not significant result (determined by confidence interval crossing 1).

Table S3. Odds ratios (OR) of adverse events (AE) as meta-analysed by route of administration.

AE	Overall OR (CI)<br>I^2, *p* Value	Subcutaneous OR (CI)<br>I^2, *p* Value	Intravenous OR (CI)<br>I^2, *p* Value	Inhaled OR (CI)<br>I^2, *p* Value	Oral OR (CI)<br>I^2, *p* Value
Site pain	**8.7**<br>(1.6, 45.9)<br>79%, 0.003	**17.5**<br>(11.1, 27.1)<br>0%, 0.915	NS<br>- , -	-	-
Flushing	**4.0**<br>(3.0, 5.3)<br>19%, 0.259	**NS**<br>- , -	-	**4.7**<br>(2.0, 11.1)<br>26%, 0.26	**4.0**<br>(2.9, 5.5)<br>27%, 0.226
Jaw Pain	**4.6**<br>(3.6, 5.8)<br>0%, 0.571	**3.1**<br>(1.6, 6.2)<br>0%, 0.929	NS<br>- , -	**2.7**<br>(1.2, 6.0)<br>14%, 0.313	**5.1**<br>(4.0, 6.7)<br>0%, 0.700
Headache	**3.6**<br>(2.4, 5.4)<br>82%, <0.001	NS<br>59%, 0.088	**6.0**<br>(1.1, 31.5)<br>- , -	**2.4**<br>(1.0, 5.7)<br>49%, 0.161	**5.4**<br>(3.5, 8.2)<br>73%, 0.001
Pain in extremity	**2.7**<br>(1.7, 3.5)<br>0%, 0.480	NS<br>- , -	**13.0**<br>(1.5, 112.3)<br>- , -	NS<br>- , -	**2.7**<br>(2.1, 3.6)<br>0%, 0.418
Diarrhoea	**2.6**<br>(2.0, 3.4)<br>46%, 0.039	**1.8**<br>(1.2, 2.9)<br>0%, 0.915	NS<br>- , -	NS<br>0%, 0.474	**3.3**<br>(2.7, 4.0)<br>0%, 0.659
Vomiting	**2.5**<br>(1.4, 4.7)<br>79%, <0.001	NS<br>12%, 0.322	**6.0**<br>(1.1, 31.5)<br>- , -	-	**2.5**<br>(1.4, 4.7)<br>85%, <0.001
Arthralgia	**1.5**<br>(1.1, 2.2)<br>0%, 0.788	-	-	-	**1.5**<br>(1.1, 2.2)<br>0%, 0.788
Nausea	**2.2**<br>(1.7, 2.8)<br>41%, 0.060	NS<br>65%, 0.091	NS<br>- , -	**1.7**<br>(1.0, 2.9)<br>0%, 0.766	**2.6**<br>(2.2, 3.2)<br>0%, 0.433
Peripheral oedema	NS<br>59%, 0.024	**3.8**<br>(1.5, 9.6)<br>- , -	-	NS<br>0%, 0.889	NS<br>45%, 0.141
Cough	NS<br>68%, 0.005	-	NS<br>- , -	**2.4**<br>(1.6, 3.5)<br>0%, 0.537	NS<br>0%, 0.668

CI = confidence interval; NS = Not significant result (determined by confidence interval crossing 1).

Table S4. Odds ratios of adverse events as meta-analysed by the concomitant therapy given during the length of the trial.

AE	Overall OR (CI) I^2, *p* Value	No Background therapy OR (CI) I^2, *p* Value	Background therapy OR (CI) I^2, *p* Value
Site pain	**8.7** (1.6, 45.9) 79%, 0.003	**8.7** (1.6, 45.9) 79%, 0.003	-
Flushing	**4.0** (3.0, 5.3) 19%, 0.259	**3.8** (2.4, 6.2) 21%, 0.281	**4.1** (2.8, 6.1) 32%, 0.195
Jaw Pain	**4.6** (3.6, 5.8) 0%, 0.571	**4.3** (2.8, 6.6) 0%, 0.588	**4.5** (3.3, 6.2) 14%, 0.326
Headache	**3.6** (2.4, 5.4) 82%, <0.001	**2.8** (1.3, 5.8) 84%, <0.001	**5.2** (3.2, 8.3) 69%, 0.012
Pain in extremity	**2.7** (1.7, 3.5) 0%, 0.480	**2.9** (1.0, 8.7) 25%, 0.265	**2.7** (2.1, 3.6) 0%, 0.424
Diarrhoea	**2.6** (2.0, 3.4) 46%, 0.039	**2.1** (1.4, 3.0) 26%, 0.228	**3.2** (2.5, 4.3) 28%, 0.237
Vomiting	**2.5** (1.4, 4.7) 79%, <0.001	NS 33%, 0.203	**3.6** (1.6, 8.2) 86%, 0.001
Arthralgia	**1.5** (1.1, 2.2) 0%, 0.788	NS - , -	**1.5** (1.0, 2.1) 0%, 0.837
Nausea	**2.2** (1.7, 2.8) 41%, 0.060	**1.8** (1.1, 2.7) 38%, 0.140	**2.6** (2.0, 3.3) 18%, 0.296
Peripheral oedema	NS 59%, 0.024	NS 53%, 0.093	NS 46%, 0.155
Cough	NS 68%, 0.005	NS 21%, 0.282	NS 81%, 0.001

CI = confidence interval; NS = Not significant result (determined by confidence interval crossing 1).

Table S5. Odds ratio (OR) of adverse events (AE) as meta-analysed by the treatment received in the one month up to the trial date.

AE	Overall OR<br>(CI)<br>I^2, *p* Value	Conventional therapy OR<br>(CI)<br>I^2, *p* Value	Epoprostenol therapy OR<br>(CI)<br>I^2, *p* Value	ERA / PDE-5i therapy OR<br>(CI)<br>I^2, *p* Value
Site pain	**8.7**<br>(1.6, 45.9)<br>79%, 0.003	NS<br><br>86%, 0.001	**21.7**<br>(1.8, 260.6)<br>- , -	-
Flushing	**4.0**<br>(3.0, 5.3)<br>19%, 0.259	**4.2**<br>(2.6, 6.6)<br>12%, 0.335	NS<br><br>- , -	**4.1**<br>(2.8, 6.1)<br>32%, 0.195
Jaw Pain	**4.6**<br>(3.6, 5.8)<br>0%, 0.571	**4.3**<br>(2.8, 6.8)<br>0%, 0.475	NS<br><br>- , -	**4.5**<br>(3.3, 6.2)<br>14%, 0.326
Headache	**3.6**<br>(2.4, 5.4)<br>82%, <0.001	**3.3**<br>(1.6, 7.0)<br>85%, <0.001	NS<br><br>- , -	**5.2**<br>(3.2, 8.3)<br>69%, 0.012
Pain in extremity	**2.7**<br>(1.7, 3.5)<br>0%, 0.480	NS<br><br>62%, 0.107	NS<br><br>- , -	**2.7**<br>(2.1, 3.6)<br>0%, 0.424
Diarrhoea	**2.6**<br>(2.0, 3.4)<br>46%, 0.039	**2.1**<br>(1.2, 2.9)<br>38%, 0.151	NS<br><br>- , -	**3.2**<br>(2.5, 4.3)<br>28%, 0.237
Vomiting	**2.5**<br>(1.4, 4.7)<br>79%, <0.001	NS<br><br>46%, 0.137	NS<br><br>- , -	**3.6**<br>(1.6, 4.7)<br>86%, 0.001
Arthralgia	**1.5**<br>(1.1, 2.2)<br>0%, 0.788	NS<br><br>- , -	-	**1.5**<br>(1.0, 2.1)<br>0%, 0.837
Nausea	**2.2**<br>(1.7, 2.8)<br>41%, 0.060	**1.8**<br>(1.3, 2.6)<br>13.1%, 0.331	NS<br>- , -	**2.6**<br>(2.0, 3.3)<br>18%, 0.269
Peripheral oedema	NS<br><br>59%, 0.024	NS<br><br>53%, 0.093	-	NS<br><br>46%, 0.155
Cough	NS<br><br>68%, 0.005	NS<br><br>21%, 0.282	-	NS<br><br>81%, 0.001

CI = confidence interval; NS = Not significant result (determined by confidence interval crossing 1).

Table S6. Weighted mean difference (WMD) of outcomes as meta-analysed by drug type.

	Overall WMD (CI) I^2, *p* Value	Treprostinil WMD (CI) I^2, *p* Value	Beraprost WMD (CI) I^2, *p* Value	Iloprost WMD (CI) I^2, *p* Value	Selexipag WMD (CI) I^2, *p* Value
6MWD	**16.3** (13.0, 19.7) 90%, <0.001	**15.9** (10.0, 21.8) 94%, <0.001	**22.8** (17.0, 28.6) 0%, 0.331	**28.5** (11.1, 46.0) 0%, 0.331	**13.0** (12.6, 13.4) 0%, 0.556
Mean Pulmonary arterial pressure	**−1.94** (−3.5, −0.3) 59%, 0.024	**NS** 60%, 0.114	**NS** 0%, 0.705	**NS** 72%, 0.058	**NS** -, -
Pulmonary vascular resistance index	**−3.1** (−5.3, −1.0) 100%, <0.001	**−4.7** (−4.8, −4.6) 0%, 0.682	**−1.5** (−1.8, −1.3) 0%, 0.465	**-**	**-**
Mixed venous saturation	**3.3** (2.9, 3.7) 3%, 0.390	NS 57%, 0.129	NS -, -	**2.1** (0.1, 4.1) -, -	NS -, -
Right arterial pressure	NS 83%, <0.001	NS 76%, 0.040	NS 0%, 1.000	NS -, -	**3.2** (1.1, 5.3) -, -
Heart rate	**−0.6** (−2.4, 1.2) 46%, 0.118	**0.3** (0.2, 0.4) -, -	NS -, -	NS 53%, 0.145	NS -, -
Cardiac index	**0.3** (0.1, 0.4) -, -	**0.2** (0.2, 0.2) 0%, 0.437	**0.2** (0.0, 0.3) 0%, 0.400	-	**0.5** (0.3, 0.7) -, -

CI = confidence interval; NS = Not significant result (determined by confidence interval crossing 1); 6MWD = six-minute walk distance.

Table S7. Standardised mean difference (SMD) of outcomes as meta-analysed by drug type.

	**Overall SMD (CI)**<br>***I*², *p* Value**	**Treprostinil SMD (CI)**<br>***I*², *p* Value**	**Beraprost SMD (CI)**<br>***I*², *p* Value**	**Iloprost SMD (CI)**<br>***I*², *p* Value**	**Selexipag SMD (CI)**<br>***I*², *p* Value**
6MWD	**1.2**<br>(0.6, 1.8)<br>98%, <0.001	**1.2**<br>(0.9, 1.6)<br>90%, <0.001	**0.9**<br>(0.0, 1.7)<br>90%, 0.002	**0.4**<br>(0.2, 0.7)<br>0%, 0.690	NS<br>98%, <0.001
Borg Dyspnea score	**−0.3**<br>(−0.4, −0.1)<br>36%, 0.133	NS<br>60%, 0.040	**−0.4**<br>(−0.6, −0.1)<br>0%, 0.356	NS<br>-, -	NS<br>-, -
Quality of Life Score	**0.2**<br>(−0.1, 0.3)<br>0%, 0.391	NS<br>62%, 0.106	NS<br>-, -	NS<br>-, -	-
Mean pulmonary arterial pressure	**NS**<br>99%, <0.001	**NS**<br>99%, <0.001	**NS**<br>0%, 0.675	**NS**<br>71%, 0.065	**NS**<br>-, -
Pulmonary vascular resistance index	**−3.6**<br>(−6.8, −0.4)<br>99%, <0.001	**−5.5**<br>(−10.1, −0.9)<br>98%, <0.001	**−1.8**<br>(−2.2, −1.5)<br>5%, 0.306	**-**	**-**
Mixed venous saturation	NS<br>99%, <0.001	NS<br>99%, <0.001	NS<br>-, -	**0.3**<br>(0.0, 0.6)<br>-, -	NS<br>-, -
Right arterial pressure	NS<br>99%, <0.001	NS<br>99%, <0.001	NS<br>0%, 0.875	NS<br>-, -	**1.0**<br>(0.2, 1.7)<br>-, -
Heart rate	NS<br>77%, 0.001	**0.40**<br>(0.22, 0.58)<br>- , -	NS<br>-, -	NS<br>55%, 0.136	NS<br>-, -
Cardiac index	NS<br>99%, <0.001	NS<br>99%, <0.001	NS<br>49%, 0.161	-	**1.1**<br>(0.4, 1.9)<br>-, -

CI = confidence interval; NS = Not significant result (determined by confidence interval crossing 1); 6MWD = six-minute walk distance.

Table S8. Standardised mean difference (SMD) of outcomes as meta-analysed by route of administration.

	**Overall SMD (CI) I^2, *p* Value**	**Subcutaneous SMD (CI) I^2, *p* Value**	**Intravenous SMD (CI) I^2, *p* Value**	**Inhaled SMD (CI) I^2, *p* Value**	**Oral SMD (CI) I^2, *p* Value**
6MWD	**1.2**<br>(0.6, 1.8)<br>98%, <0.001	**1.1**<br>(0.6, 1.7)<br>47%, 0.152	**0.7**<br>(0.1, 1.4)<br>-, -	NS<br>96%, <0.001	**1.4**<br>(0.4, 2.4)<br>99%, <0.001
Borg Dyspnea score	**−0.3**<br>(−0.4, −0.1)<br>36%, 0.133	NS<br>17%, 0.300	**−1.0**<br>(−1.6, −0.3)<br>-, -	NS<br>41%, 0.193	**−0.3**<br>(−0.6, −0.1)<br>0%, 0.459
Quality of Life Score	**0.2**<br>(0.1, 0.3)<br>0%, 0.391	NS<br>-, -	-	**0.3**<br>(0.1, 0.6)<br>9%, 0.293	NS<br>-, -
Mean pulmonary arterial pressure	**NS**<br>99%, <0.001	NS<br>99%, <0.001	-	**NS**<br>71%, 0.065	**−0.26**<br>(−0.53, 0.00)<br>0%, 0.502
Pulmonary vascular resistance index	**−3.6**<br>(−6.8, −0.4)<br>99%, <0.001	**−5.5**<br>(−10.1, −0.9)<br>98%, <0.001	-	**-**	**−1.8**<br>(−2.2, −1.5)<br>5%, 0.306
Mixed venous saturation	NS<br>99%, <0.001	NS<br>99%, <0.001	-	**0.3**<br>(0.0, 0.6)<br>-, -	NS<br>0%, 0.588
Right arterial pressure	NS<br>99%, <0.001	NS<br>99%, <0.001	-	NS<br>-, -	NS<br>99%, 0.027
Heart rate	NS<br>77%, 0.001	**0.4**<br>(0.2, 0.6)<br>-, -	-	NS<br>55%, 0.136	NS<br>57%, 0.126
Cardiac index	NS<br>99%, <0.001	NS<br>99%, <0.001	-	-	NS<br>66%, 0.052

CI = confidence interval; NS = Not significant result (determined by confidence interval crossing 1); 6MWD = six-minute walk distance.

Table S9. Weighted mean difference (WMD) of outcomes as meta-analysed by route of administration.

	**Overall WMD (CI)** *I^2, p* **Value**	**Subcutaneous WMD (CI)** *I^2, p* **Value**	**Intravenous WMD (CI)** *I^2, p* **Value**	**Inhaled WMD (CI)** *I^2, p* **Value**	**Oral WMD (CI)** *I^2, p* **Value**
	**16.3**	NS	**93.0**	**19.5**	**15.3**
6MWD	(13.0, 19.7)		(8.3, 178)	(15.5, 23.5)	(11.1, 19.5)
	90%, <0.001	87%, <0.001	-, -	3%, 0.356	93%, <0.001
	**−1.94**	**NS**		**NS**	**NS**
Mean pulmonary arterial pressure	(−3.5, −0.3)		**-**		
	59%, 0.024	60%, 0.114		72%, 0.058	0%, 0.460
	**−3.1**	**−4.7**			**−1.5**
Pulmonary vascular resistance index	(−5.3, −1.0)	(−4.8, −4.6)	**-**	**-**	(−1.8, −1.3)
	100%, <0.001	0%, 0.682			0%, 0.465
	**3.3**	NS		**0.3**	NS
Mixed venous saturation	(2.9, 3.7)		-	(0.0, 0.6)	
	3%, 0.390	99%, <0.001		-, -	99%, <0.001
	NS	NS		NS	NS
Right arterial pressure			-		
	83%, <0.001	76%, 0.040		-, -	75%, 0.018
	NS	**0.3**		NS	NS
Heart rate		(0.2, 0.4)	-		
	46%, 0.118	-, -		53%, 0.145	62%, 0.107
	**0.3**	**0.2**			**0.3**
Cardiac index	(0.1, 0.4)	(0.2, 0.2)	-	-	(0.0, 0.5)
	60%, 0.041	0%, 0.437			70%, 0.036

CI = confidence interval; NS = Not significant result (determined by confidence interval crossing 1); 6MWD = six-minute walk distance.

Table S10. Odds ratios (OR) of outcomes as meta-analysed by drug type.

	**Overall OR (CI)** I^2, *p* **Value**	**Treprostinil OR (CI)** I^2, *p* **Value**	**Beraprost OR (CI)** I^2, *p* **Value**	**Iloprost OR (CI)** I^2, *p* **Value**	**Selexipag OR (CI)** I^2, *p* **Value**
Discontinuation from adverse event	NS<br>61%, 0.009	NS<br>74%, 0.002	NS<br>-, -	NS<br>-, -	2.2<br>(1.5, 3.3)<br>-, -
Clinical deterioration	0.4<br>(0.2, 0.8)<br>47%, 0.080	NS<br>65%, 0.036	-	NS<br>-, -	0.3<br>(0.2, 0.5)<br>0%, 0.440
Fatalities	NS<br>14%, 0.319	NS<br>0%, 0.511	NS<br>-, -	NS<br>-, -	NS<br>-, -
Lung transplant	NS<br>0%, 0.870	NS<br>0%, 0.610	-	-	NS<br>-, -
Rescue therapy	NS<br>0%, 0.695	NS<br>0%, 0.488	NS<br>-, -	NS<br>-, -	NS<br>-, -
Right ventricular heart failure	NS<br>0%, 0.485	NS<br>0%, 0.380	NS<br>-, -	NS<br>-, -	-

CI = confidence interval; NS = Not significant result (determined by confidence interval crossing 1).

Table S11. Odds ratios (OR) of outcomes as meta-analysed by route of administration.

	Overall OR (CI) I^2, *p* Value	Subcutaneous OR (CI) I^2, *p* Value	Intravenous OR (CI) I^2, *p* Value	Inhaled OR (CI) I^2, *p* Value	Oral OR (CI) I^2, *p* Value
Discontinuation from adverse event	NS<br>61%, 0.009	NS<br>89%, <0.001	NS<br>-, -	NS<br>0%, 0.703	**2.1**<br>(1.5, 3.0)<br>0%, 0.743
Clinical deterioration	**0.4**<br>(0.2, 0.8)<br>47%, 0.080	NS<br>88%, 0.004	-	NS<br>-, -	**0.4**<br>(0.3, 0.5)<br>0%, 0.485
Fatalities	NS<br>14%, 0.319	NS<br>-, -	NS<br>-, -	NS<br>0%, 0.863	NS<br>0%, 0.691
Lung transplant	NS<br>0%, 0.870	NS<br>-, -	-	-	NS<br>0%, 0.712
Rescue therapy	NS<br>0%, 0.695	NS<br>0%, 0.866	NS<br>-, -	NS<br>-, -	NS<br>0%, 0.448
Right ventricular heart failure	NS<br>0%, 0.485	NS<br>-, -	-	NS<br>4%, 0.309	NS<br>36%, 0.211

CI = confidence interval; NS = Not significant result (determined by confidence interval crossing 1).

Table S12. Meta-regression for outcomes.

	Meta-Regression	Coefficient	Standard Error	*p*-Value
Clinical deterioration	Mean age	0.0590	0.2755	0.844
	% female	0.1150	0.2858	0.714
	% IPAH	0.0092	0.1605	0.958
Adverse event discontinuation	Mean age	0.0780	0.1065	0.491
	% female	−0.0092	0.0839	0.916
	% IPAH	0.0897	0.0789	0.319
Fatalities	Mean age	−0.0649	0.0382	0.133
	% female	0.0779	0.3455	0.064
	% IPAH	0.0217	0.0312	0.517
6MWD (WMD)	Mean age	−0.7538	0.6187	0.249
	% female	−0.6544	0.4702	0.191
	% IPAH	0.0094	0.4465	0.984
6MWD (SMD)	Mean age	−0.0045	0.0411	0.915
	% female	0.0170	0.0323	0.609
	% IPAH	0.0300	0.0290	0.328
Borg (WMD)	Mean age	0.1440	0.0881	0.350
	% female	0.0729	0.0666	0.472
	% IPAH	0.0606	0.0577	0.485
Borg (SMD)	Mean age	0.0511	0.0266	0.306
	% female	0.6314	0.0329	0.306
	% IPAH	0.0368	0.0260	0.392
Rescue therapy	Mean age	0.1580	0.1148	0.262
	% female	0.0084	0.1104	0.944
	% IPAH	0.0828	0.0680	0.310

IPAH = idiopathic pulmonary arterial hypertension. 6MWD = six-minute walk distance; WMD = weighted mean difference; SMD = standardised mean difference.

Table S13. Eggers and begs values tests detecting publication bias on overall groups.

	Number of Studies	Eggers	Beggs	Funnel Plot Symmetry
6MWD (WMD)	14	0.119	0.189	NSy
6MWD (SMD)	14	0.250	0.584	NSy
Borg (WMD)	6	0.914	0.707	
Borg (SMD)	9	0.716	1.000	Sy
Mean pulmonary arterial pressure (WMD)	7	0.248	0.881	NSy
Mean pulmonary arterial pressure (SMD)	7	0.955	0.176	NSy
Pulmonary vascular resistance index (WMD)	4	0.362	0.999	NSy
Pulmonary vascular resistance index (SMD)	4	0.897	0.497	NSy
Mixed venous saturation (WMD)	5	0.327	0.806	NSy
Mixed venous saturation (SMD)	5	0.834	0.462	NSy
Right arterial pressure (WMD)	6	0.063	0.844	NSy
Right arterial pressure (SMD)	6	0.879	0.260	NSy
Cardiac index (WMD)	5	0.395	0.462	NSy
Cardiac index (SMD)	5	0.527	1.000	NSy
Heart Rate (WMD)	5	0.262	0.086	NSy
Heart Rate (SMD)	5	0.126	0.462	NSy
Clinical deterioration	7	0.727	0.368	
Fatalities	11	0.035	0.251	NSy
Rescue therapy	8	0.240	0.711	
Discontinuation due to AE	9	0.457	0.466	Sy

6MWD = six-minute walk distance; WMD = weighted mean difference; SMD = standardised mean difference; AE = adverse event; Sy = symmetrical; NSy = not symmetrical.

Reference

1. Research Triangle Park, United Therapeutics, MA, U. Remodulin® (treprostinil) Injection, for subcutaneous or intravenous use. Available online: https://www.remodulin.com/downloads/remodulin-prescribinginformation.pdf (accessed on 1 November 2015).
2. United Therapeutics Corp. *Tyvaso (treprostinil) prescribing information*; Research triangle park, NC, 2009. Available online: https://www.accessdata.fda.gov/drugsatfda_docs/label/2009/022387LBL.pdf (accessed on 1 July 2017)
3. United Therapeutics Corp. *Full prescibing information: Orenitram (TM) Extended release tablets for oral administration*; Research Triangle Park, 2013. Available online: https://www.accessdata.fda.gov/drugsatfda_docs/label/2013/203496s000lbl.pdf (accessed on 1 July 2017)
4. European Medicines Agency *Uptravi (selexipag): Summary of Product characteristics*; London, 2016. Available online:http://www.ema.europa.eu/docs/en_GB/document_library/EPAR_-_Product_Information/human/003774/WC500207173.pdf (accessed on 1 July 2017)
5. Beraprost pharmacology Available online: https://www.drugbank.ca/drugs/DB05229 (accessed on 1 July 2017).
6. European Medicines Agency *Ventavis—Scientific Discussion*; London, 2004. Available online: http://www.ema.europa.eu/docs/en_GB/document_library/EPAR_-_Scientific_Discussion/human/000474/WC500048688.pdf (accessed on 1 July 2017)
